# AxiWorm: a new tool using YOLOv5 to test antiparasitic drugs against *Trichinella spiralis*

**DOI:** 10.1186/s13071-025-06664-8

**Published:** 2025-02-02

**Authors:** Javier Sánchez-Montejo, Miguel Marín, María Alejandra Villamizar-Monsalve, María del Carmen Vieira, Belén Vicente, Rafael Peláez, Julio López-Abán, Antonio Muro

**Affiliations:** 1 Infectious and Tropical Diseases Research Group (E-INTRO), Biomedical Research Institute of Salamanca-Research Centre for Tropical Diseases at the University of Salamanca (IBSAL-CIETUS), Faculty of Pharmacy, University of Salamanca, 37007 Salamanca, Spain; 2 Department of Pharmaceutical Sciences, Biomedical Research Institute of Salamanca-Research Centre for Tropical Diseases at the University of Salamanca (IBSAL-CIETUS), Faculty of Pharmacy, University of Salamanca, 37007 Salamanca, Spain

**Keywords:** *Trichinella spiralis*, YOLOv5, Computer vision, Drug screening, AxiWorm

## Abstract

**Background-Objective:**

*Trichinella spiralis* drug development and control need an objective high throughput system to assess first stage larvae (L1) viability. YOLOv5 is an image recognition tool easily trained to count muscular first stage larvae (L1) and recognize morphological differences. Here we developed a semi-automated system based on YOLOv5 to capture photographs of 96 well microplates and use them for L1 count and morphological damage evaluation after experimental drug treatments.

**Material and methods:**

Morphological properties were used to distinguish L1 from debris after pepsin muscle digestion and distinguish healthy (serpentine) or damaged (coiled) L1s after 72 h untreated or treated with albendazole or mebendazole cultures. An AxiDraw robotic arm with a smartphone was used to scan 96 well microplates and store photographs. Images of L1 were manually annotated, and augmented based on exposure, bounding, blur, noise, and mosaicism.

**Results:**

A total of 1309 photographs were obtained that after L1 labeling and data augmentation gave 27478 images. The final dataset of 12571 healthy and 14907 affected L1s was used for training, testing, and validating in a ratio of 70/20/10 respectively. A correlation of 92% was found in a blinded comparison with bare-eye assessment by experienced technicians.

**Conclusion:**

YOLOv5 is capable of accurately counting and distinguishing between healthy and affected L1s, thus improving the performance of the assessment of meat inspection and potential new drugs.

**Graphical Abstract:**

**Figure:**
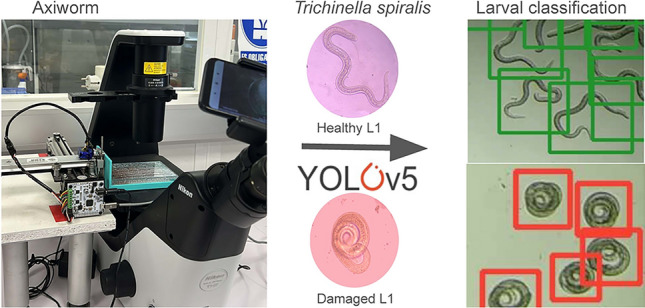

## Background

*Trichinella spiralis* is a globally distributed parasitic nematode causing trichinellosis, a zoonotic foodborne disease affecting both humans and animals [1]. Domestic and wild animals, particularly carnivores, serve as reservoirs, facilitating the persistence of the parasite and consequently threatening both livestock and human health [2]. This is especially significant given the ongoing public health concern surrounding *T. spiralis* infections, notably in regions where the consumption of undercooked pork or game meat of wild boar, bear, deer, moose, or walrus is prevalent [3–6]. Approximately 10,000 cases of trichinellosis are reported annually worldwide, with significant morbidity and mortality [7]. Clinical manifestations such as nausea, vomiting, abdominal pain, and diarrhea start within 12–48 h after eating contaminated meat when first-stage larvae (L1) develop into adults. Later, L1 migration results in fever, malaise, chest pain, muscle pain, and skin rashes within 5 to 7 days. Muscle pain and fever may persist for 1 to 6 weeks. Encysted larvae may lead to injuries ranging from slight muscle damage to severe complications such as sepsis [1, 5]. In animals, *T. spiralis* infections reduce productivity and increase susceptibility to other diseases, particularly in swine [8, 9]. The economic impact of *T. spiralis* infections encompasses direct costs associated with control measures, slaughterhouse seizures, and trade restrictions [10, 11].

Current treatment options for *T. spiralis* infections primarily involve using benzimidazoles such as albendazole or oxfendazole. These drugs have limited efficacy against encysted larvae, and therefore new alternatives are needed [12, 13]. L1 cultures are used in in vitro drug screening, where L1 viability is associated with mobility, morphological shapes, and integrity of larvae. Promising candidates could be used against experimental infections in a trichinellosis mouse model. In both cases, a trained observer must conduct extensive optical microscope sessions to count healthy and damaged larvae or determine the number of larvae per gram of muscle [14, 15]. In the development pipeline of new drugs, objective high-throughput techniques are needed to screen large numbers of candidates. A computer vision model for larval categorization and count estimation systems could be useful to avoid limitations such as subjectivity and interassay variability and to improve repeatability, accuracy, and time consumption of the analysis. These tasks could be performed by AI (Artificial Intelligence) applications based on deep-learning-based image recognition powered by convolutional neural networks (CNNs) [16]. A wide range of domains has been revolutionized by object recognition: autonomous vehicles, robotics, security surveillance, or medical imaging [17]. In parasitology, it has been used for helminth detection, facilitating automated egg scanning from soil-transmitted species like *Ascaris lumbricoides*, *Trichuris trichiura* [18], or hookworms based on their sizes, shapes, and texture features. Furthermore, it can assess viability based on morphology and motility, extending to nematodes like *Brugia malayi* and *Dirofilaria immitis* [19]. Simpler architectures like YOLO (You Only Look Once) have demonstrated remarkable performance in detecting general objects [20]. These models operate efficiently without requiring extensive knowledge or computational power. YOLOv5-based automatic recognition algorithms have already been employed in detecting parasitic eggs in human feces [21] and *Onchomelania* snails, an intermediate host of *Schistosoma japonicum* [22]. These studies could be further improved by adding an automated image acquisition system like the one proposed in this study, which reduces the time the plates stay out of the incubators and therefore the biological variation due to culture handling.

We describe the development of a semi-automatic image analysis based in YOLOv5 system linked to a Do-It-Yourself image acquisition apparatus (AxiWorm) for the assessment of first-stage larvae (L1) of *Trichinella spiralis*. This will be used to evaluate the antiparasitic activity of putative drugs in in vitro cultures and to count L1 larvae per gram of skeletal muscle after experimental treatment. YOLOv5 is an image recognition model requiring a middle level of programming skill and with limited computational power useful in a parasitology research laboratory.

## Methods

### *Trichinella spiralis* life cycle in CD1 mice

Seven-week-old, outbred SPF CD1 female mice (Charles River Laboratories, Lyon, France) were used to maintain the *T. spiralis* life cycle and for in vivo drug testing. Animal procedures complied with Spanish (RD 53/2013) and European Union (Di 2010/63/CE) regulations regarding animal experimentation for the protection of laboratory animals. The accredited Animal Experimentation Facilities (registration number: PAE/SA/001) of the University of Salamanca (USAL) were used for the procedures. The USAL’s Research Ethics Committee approved the procedures used in this study (ref. CEI 1080 and CEI 1062). All efforts were made to minimize animal suffering. Animals were maintained in USAL animal facilities in standard polycarbonate cages, with controlled 12 h light and dark periods, temperature of 23–25 °C, and food and water ad libitum. The *T. spiralis* strain used in this study was originally isolated in 1962 from a naturally infected wildcat (*Felis silvestris* MFEL/SP62/ISS48) from Pola de Lena (Asturias, Spain) [23]. It was maintained in Swiss mice (CD-1) in passes every 6 months with oral doses of 600 first-stage muscular larvae.

### First-stage larvae (L1) culture and treatment

Mice infected with 600 L1 at least 40 days post-infection were killed, and their carcasses were extracted in aseptic conditions. Carcasses were minced and digested in a solution with 0.7% HCl, 0.5% pepsin, and 0.85% NaCl for 90 min, 37 ºC, in agitation. Digested carcasses were filtered through a gaze maze, and L1s were collected in a sedimentation vase. The supernatant was removed by aspiration, and L1s were washed three times with a sterile 0.85% NaCl solution. L1s were suspended in lysogeny broth (LB) (10 g/l NaCl, 10 g/l peptone, 5 g/l yeast extract) and placed into 96-well plates at a ratio of 30–50 L1/well in a final volume of 200 µl. Albendazole and mebendazole were used as reference drugs at 20 µM. Both LB and DMSO 1% were used as untreated controls for the assay. The external wells of the plates were filled with sterile water to avoid a border effect. Plates were incubated at 37 ºC for up to 72 h before microscope assessment and image acquisition.

### Image capture, labeling, and data augmentation

**Fig. 1:**
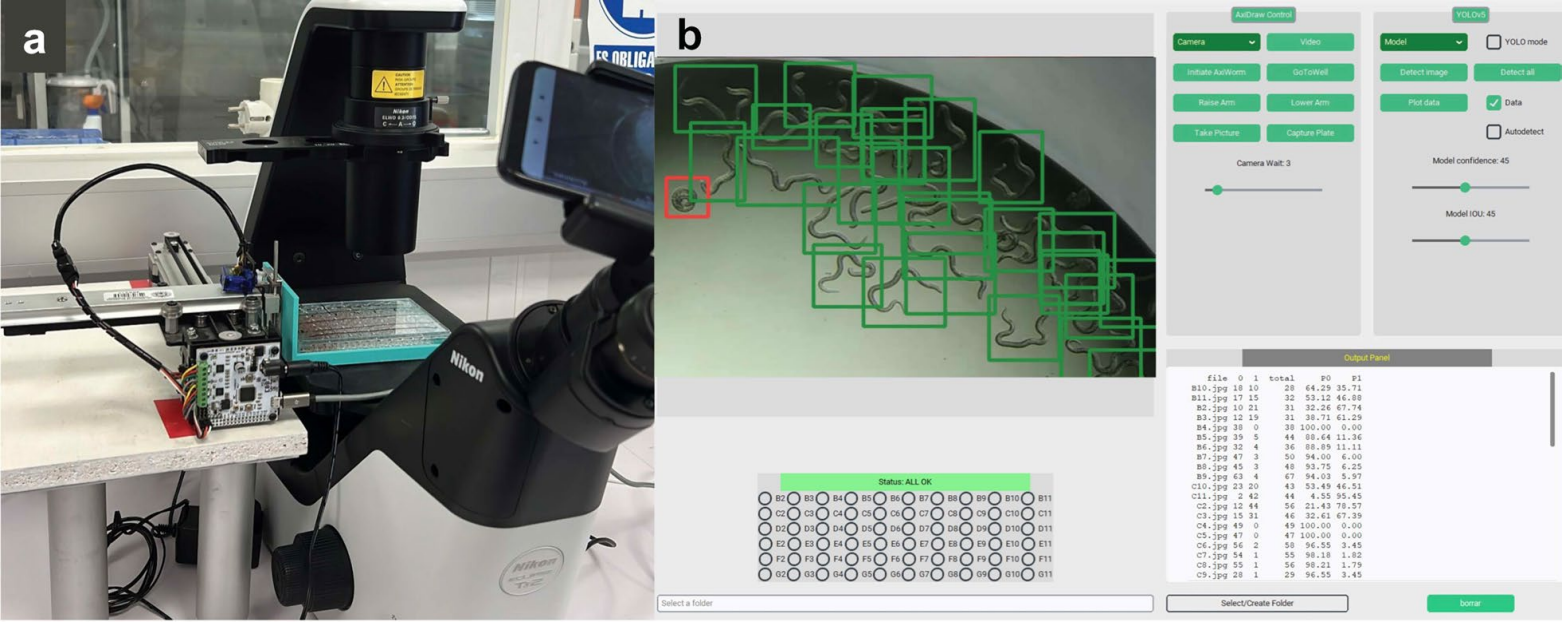
Images showing the disposition of the AxiWorm system. **a** Hardware of AxiWorm with the robotic arm over the microscope. **b** Screenshot of the interface developed to handle the system

We developed a system for image acquisition (AxiWorm) assisted by a robotic arm using an AxiDraw minikit (Evil Mad Scientist, Sunnyvale, Ca, USA) modified using 3D-printed parts to fit 96-well plates. The arm was coupled to an inverted microscope Eclipse Ts2 (Nikon, Tokyo, Japan) and a Redmi 7 smartphone (Xiaomi, Haidian, China) (Fig. 1) that imaged the entire bottom of the well using customary python scripts. Every cultured L1 was manually labeled in all captured photographs using the LabelImg python library (Fig. 1).

**Fig. 2:**
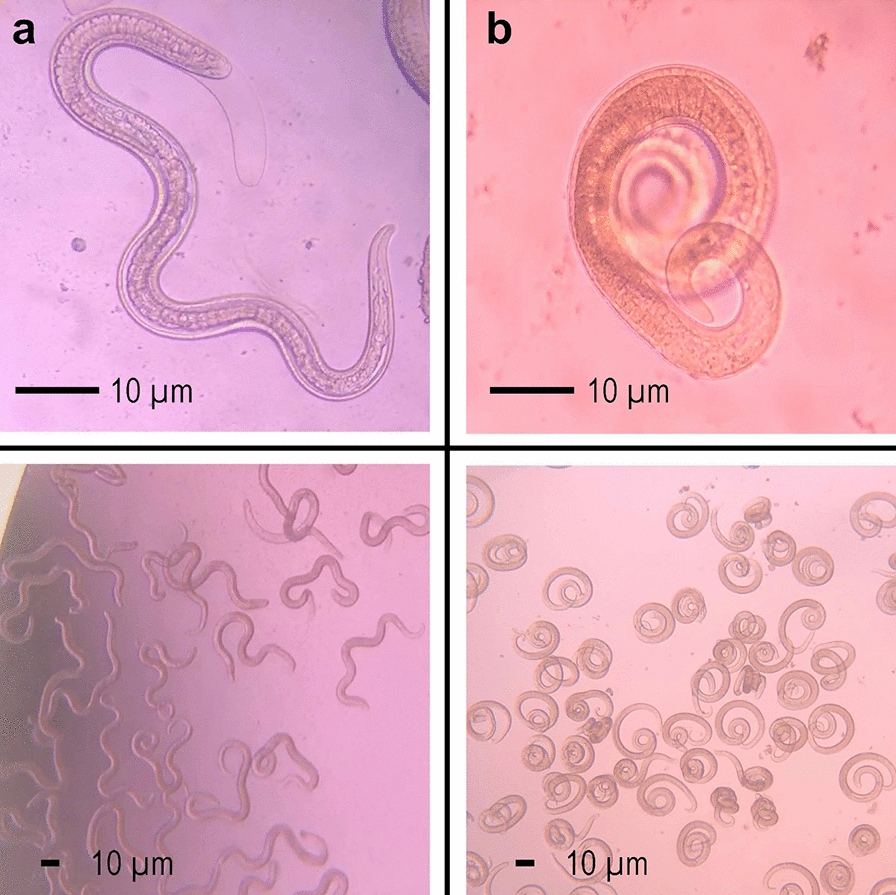
Class definition of the cultured first-stage larvae used in the model. **a** Healthy larvae showing a relaxed or loose shape. **b** Larvae damaged by the treatment showing a coiled shape

Photographs could include debris from culture media or digestion rests that remained unlabeled. Every L1 in the images was classified as loose or serpentine in shape and considered “healthy larvae” with integrity of neuromuscular function (Fig. 2a) or as “damaged larvae” with coiled shapes indicating loss of muscular tone which perturbs free movement (Fig. 2b). A total of 1309 photographs, containing 109 null images, were annotated, and the dataset was split into training, validation, and test sets with a 70/20/10 ratio. Data augmentation was applied to the training set using the web platform Roboflow [24] applying random changes in brightness (± 25%), exposure (± 25%), horizontal bounding box flip, bounding box rotation (± 15%), blur (up to 10 px), noise (up to 2%), and mosaicism.

### YOLOv5 model training and validation

The YOLOv5 (Ultralytics) model was trained without pre-trained weights with patience set to 25 and batch size to 16. From the available model sizes, we selected YOLOv5x and used default hyperparameter settings. Input images were scaled to 640 × 640 pixels. Training processing was performed on a Windows 10 computer with Intel (R) Core i7-13700KF 3.40 GHz (16 × core), RAM 32 GB DDR5 5600 MHz 2 × 16 GB CL36, and a GeForce RTX 3060 graphics card with 12 GB of dedicated RAM GDDR6 (NVIDIA, Santa Clara, CA, USA) using PyTorch 2.0.1 (Linux Foundation, San Francisco, CA, USA) with CUDA 12.2.r12.2 (NVIDIA).

The internal validation of the model was performed using the “val function” included with the package using the images reserved for that purpose. Mean average precision (mAP) is used to evaluate object detection models involving the number of classes and the average precision for each class. Also, recall [true positive/(true positive plus false positive)] and precision [true positive/(true positive plus false negative)] values were calculated. The loss value was used to reflect the error between the final prediction result and the actual true value for box placement, probability of the object existing in the region of interest, and classification accuracy.

### External validation for larval recognition and classification

For external validation, 100 new randomly selected images were augmented three times by random rotations and mirroring. The 300 resulting images were evaluated by three independent technicians and the YOLOv5 model using a confidence threshold of 0.4. The observers were given instructions to read 300 images as in a routine laboratory assay, counting and classifying the larvae in both groups: healthy or damaged. Then, the produced numbers were analyzed to account for inter- and intraobserver variability and to compare human and YOLOv5 results.

### AxiWorm validation for larval burden determination in an animal trichinellosis model

In vivo assay of antiparasitic drugs in *T. spiralis* requires the determination of muscular larvae burden by counting the number of larvae per gram of tissue. To assess whether the model could be used to reduce the laborious task of counting the larvae, 12 mice were divided into two groups of six animals and infected with 600 L1 by oral gavage [25]. After infection, one group was treated with 50 µg albendazole per gram of mice at days 13, 14, and 15 post-infection, and the other was kept untreated as an infection control. At day 40 post-infection, the mice were killed, and the back limbs were weighed and ground using two 40-s, 6 m/s cycles in a FastPrep system with 6/7 steel beads. Every sample was individually digested in pepsin as above and placed across a whole row of wells in a 96-well plate. Then, images were captured using the AxiWorm system, and the total number of larvae in the images was counted both by a human observer twice and averaged and by the YOLOv5 model using a confidence threshold of 0.4 to compare both counting techniques.

### Data analysis

Statistical analysis was performed using R Studio software [26] with non-parametric tests (Kruskal–Wallis), adjusted by Holm and pairwise comparisons by Dunn test. *P*-values < 0.05 were considered statistically significant. Coefficients of variation (CVs) were calculated as the standard deviation of the measurements divided by the mean. Tendency lines and confidence intervals were calculated with the smooth function of “ggplot2” and a linear model. Plots and figures were made using “ggplot2” and “ggstatsplots” [27]. Lastly, we used the Bland-Altman plot to identify biases, and the relative precision of the compared measurement techniques was calculated using the “BlandAltmanLeh” package [28].

## Results

### Image capture and labeling

A total of 1309 photographs, containing 109 null images, were automatically captured using AxiWorm. After applying the augmentation, the dataset consisted of 11,855 labeled photographs. A sum of 27,478 larvae were manually labeled. There was an average of 21 images per photograph. A total of 12,571 of the annotated L1 images were labeled as healthy and 14,907 as damaged.

### Larval recognition classes and internal validation

**Fig. 3:**
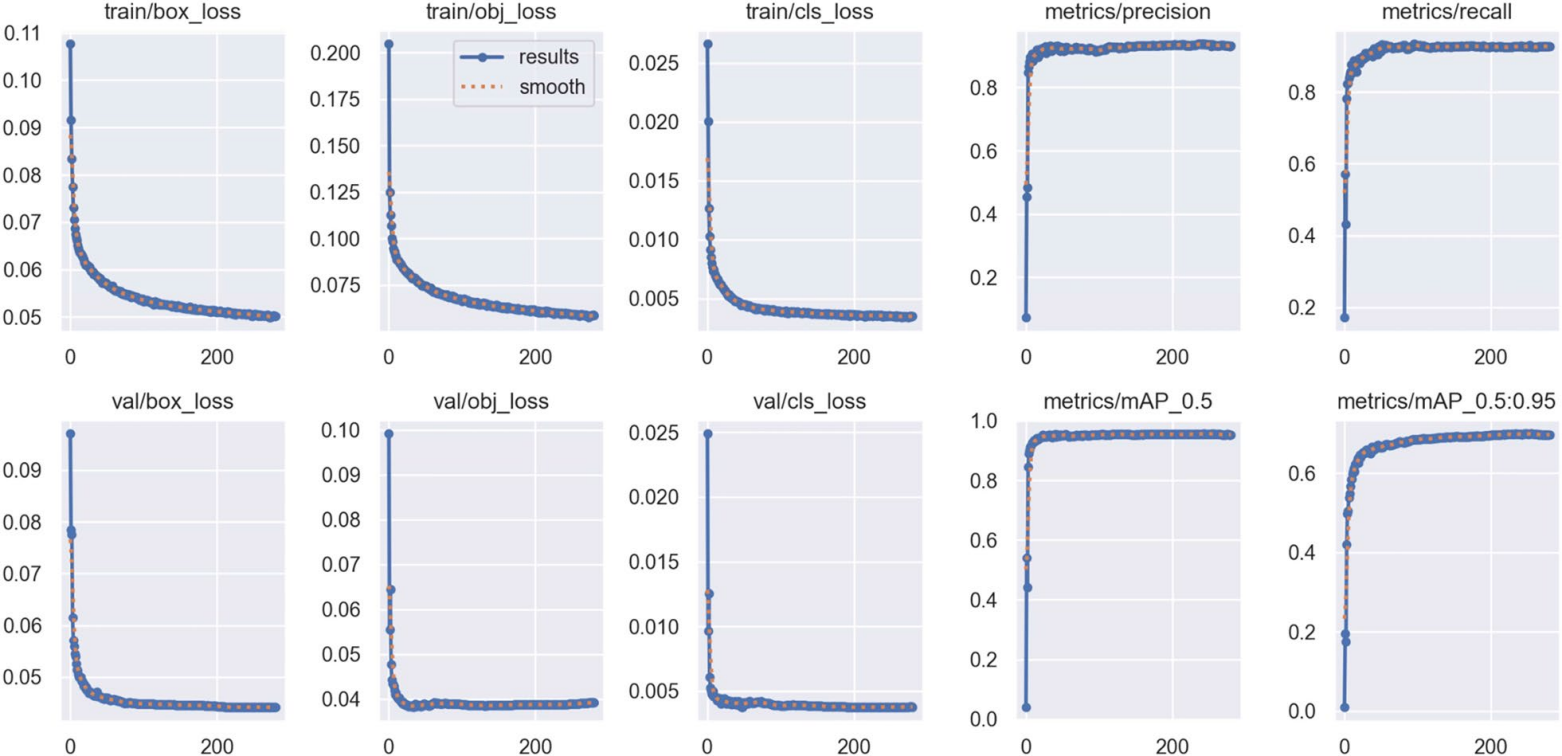
Visual representation of the scale of change (0 to 1) in the evaluation metrics across training epochs in the training and validation set

The model was trained for a total of 280 epochs that took approximately 9 days of computational power. Most of the improvement in the model occurs during the initial epochs reaching a mean average precision at confidence 0.5 (mAP50) over 0.90 in epoch 5. Also, precision and recall both reached high levels in early epochs and stood stable during the rest of the training. However, the metric for mAP50:95, which refers to the mean average precision across confidences 0.5 to 0.95, kept improving slowly over 255 epochs after which the training was stopped by the patience settings. Final values of mean precision peaked at 0.957 for mAP50 and 0.7 for mAP50:95. In both cases, the individual values of mAPs for damaged larvae were slightly higher than for healthy ones (Fig. 3).

**Fig. 4:**
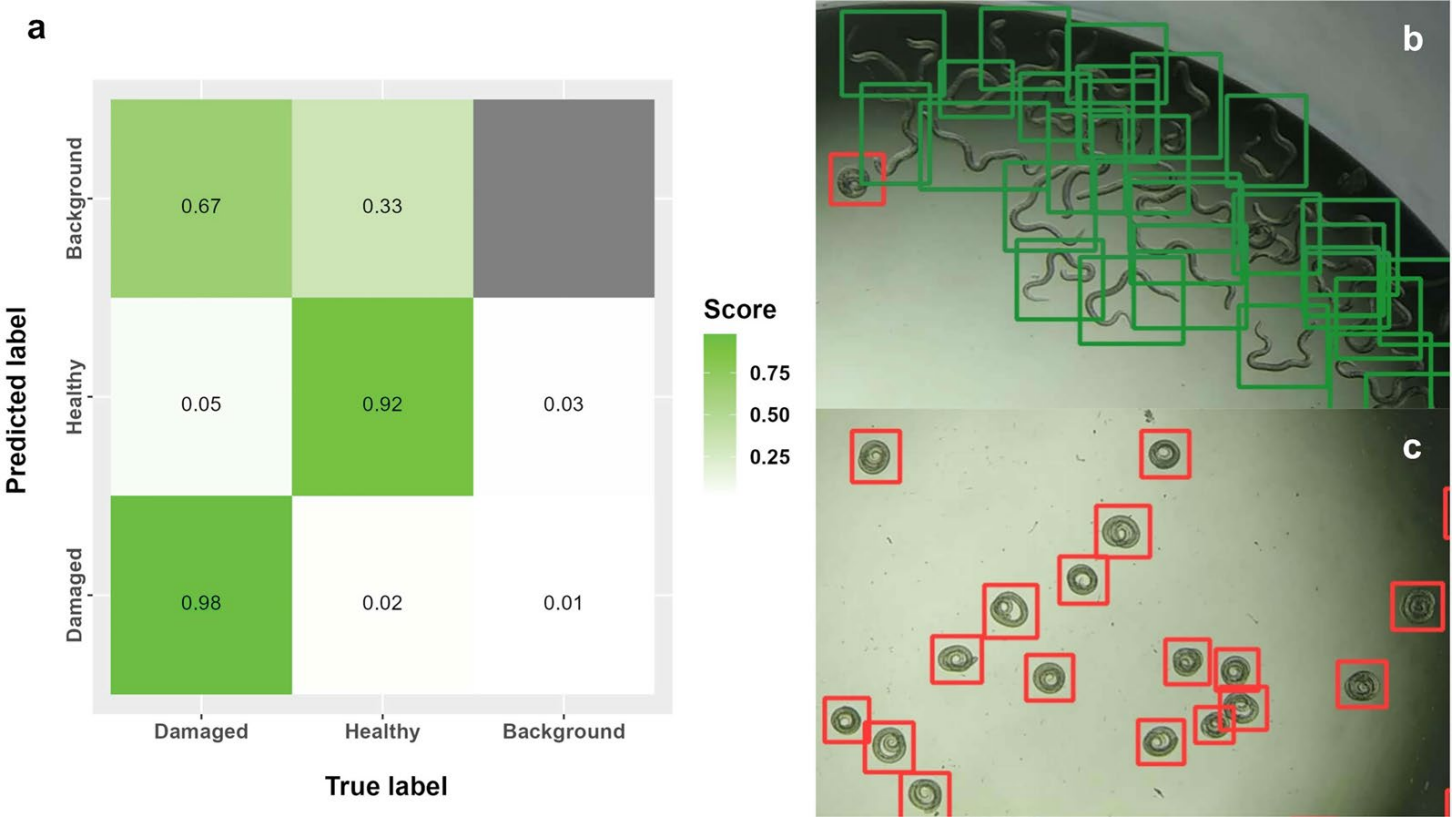
Performance of the model by internal YOLOv5 validation. **a** Confusion matrix indicating the distribution of true-positive and false-negative predicted larvae. **b** Image showing the application of the model in a plate with healthy larvae. **c** Image showing the application of the model but with damaged larvae by albendazole

The internal validation of the model was performed with the images from the original validation set. The validation yielded a precision value of 0.937 and a recall of 0.927. The model classified 98% of the damaged larvae and 92% of the healthy larvae predicted in the images correctly (Fig. 4).

### External model validation of larval recognition

**Fig. 5:**
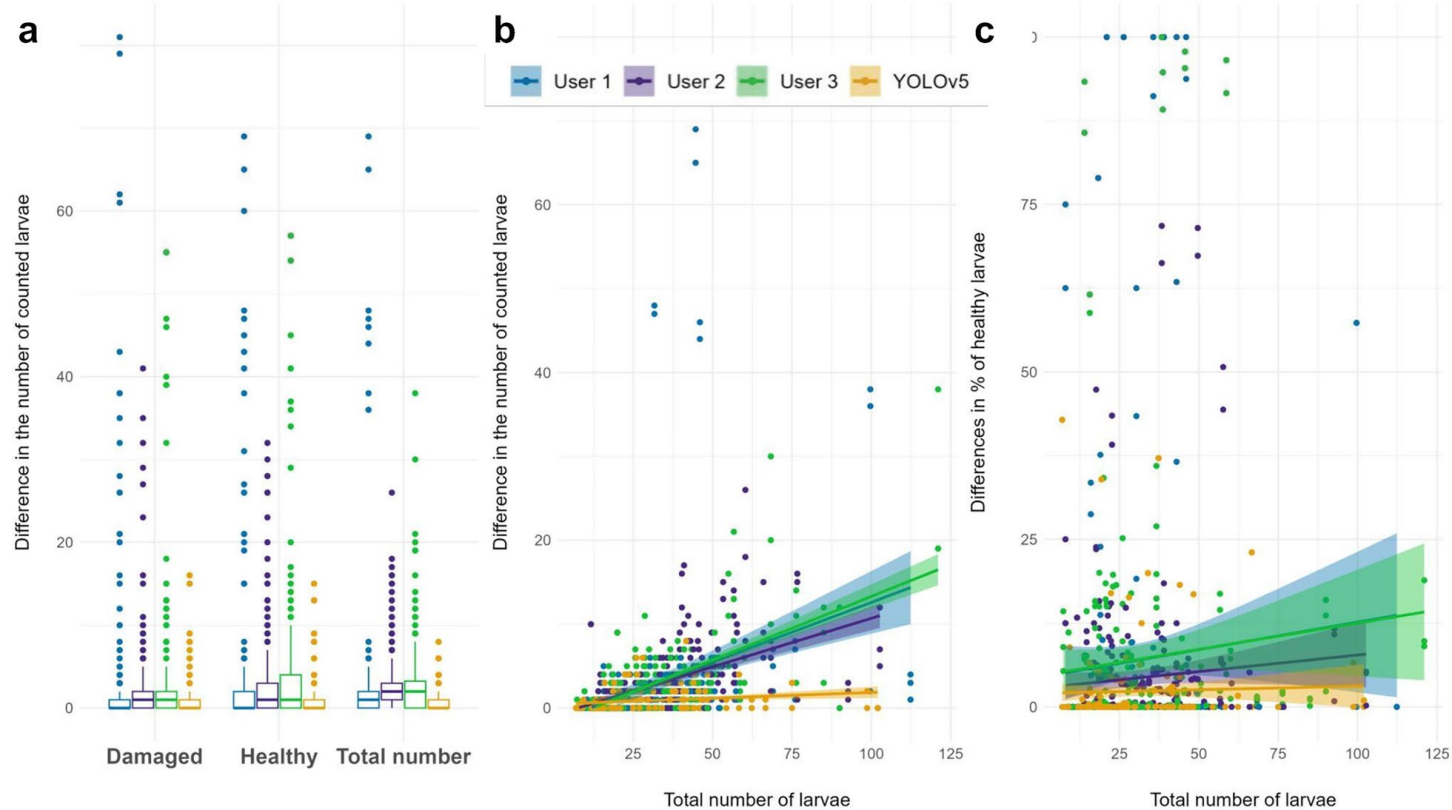
**a** Graphical representation of the differences in the numbers of counted worms between replicates by each observer for each L1 class and for the total number of larvae. **b** Linear model of the difference among the three measurements of each observer against the total number of larvae counted. **c** Linear model of the difference in the average percentage of healthy larvae predicted for the three images against the total number of larvae counted

After training the model to a point where the internal validation was up to the required standard, we compared the measurements of our model with the ones performed by three human observers. In the repeated measurements, the differences in the number of larvae predicted by the model are lower than when made by human observers (Fig. 5A). Furthermore, Fig. 5B shows that for the human observers, the differences in L1 counts greatly increase with the number of larvae, while for the model the slope is not as steep. This variability further increases when larvae are not just counted but classified and the percentage of healthy larvae is accounted for (Fig. 5C).

**Fig. 6:**
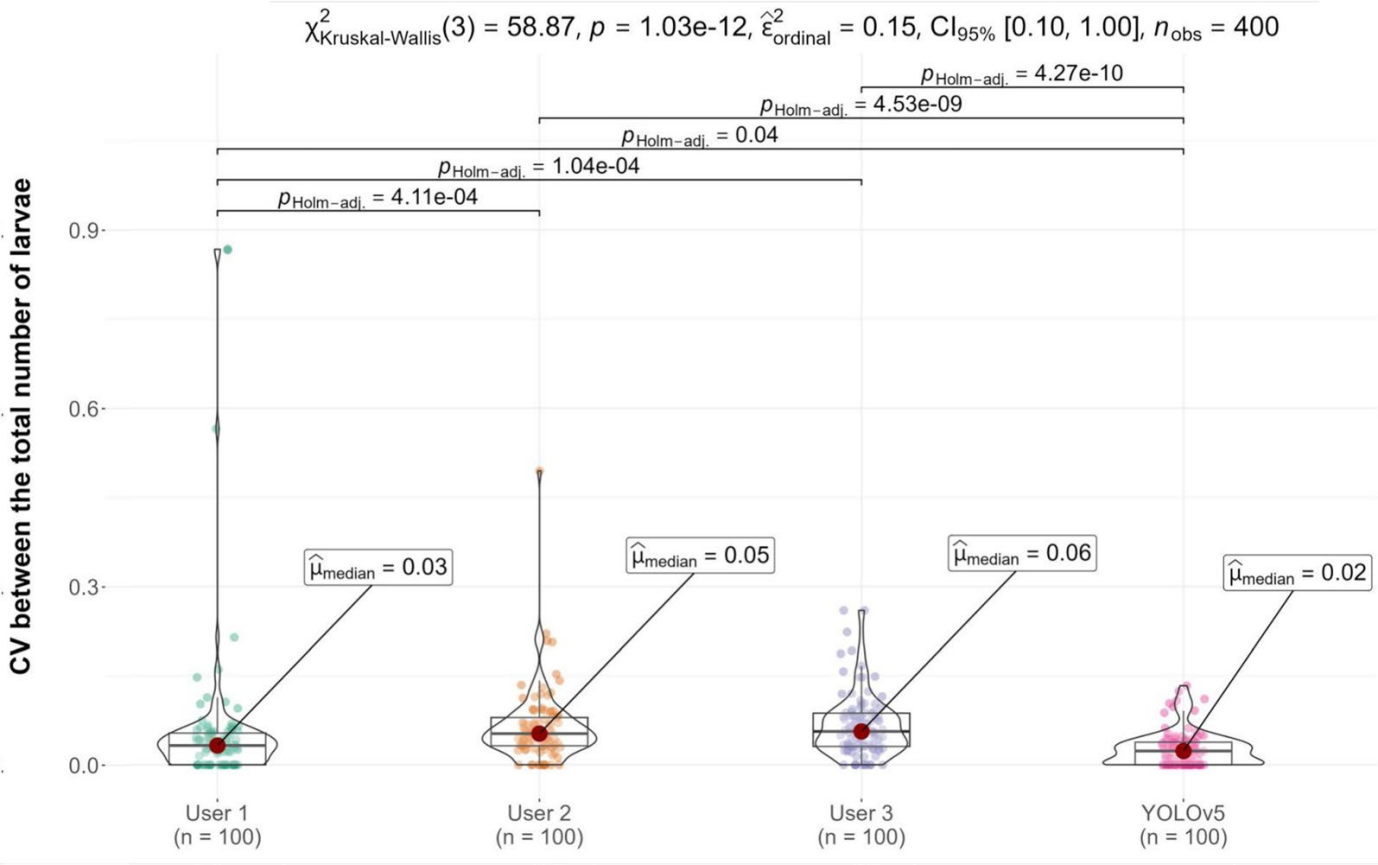
Coefficient of variation (CV) estimates of the measurements of the total number of larvae. Statistically significant (P_adj_ < 0.05) comparisons are shown by pairwise Dunn test

Using the different measurements of the total number of larvae for each image, the coefficient of variation (CV) was calculated for each observer and set of images. Then, the measurements were compared by Kruskal-Wallis for differences in median variation giving a statistically significant difference (X^2^(3) = 57.87, *p* = 1.03e−12). Furthermore, pairwise comparisons were performed by Dunn test. All the human observers’ CVs were significantly higher than those for the YOLOv5 model (Fig. 6). Also, the CV scores for the human observers varied significantly between each other.

**Fig. 7:**
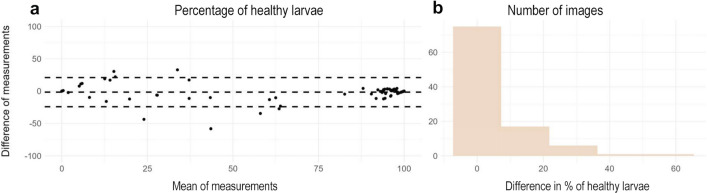
**a** Bland-Altman plot comparing the predictions made by the mean of the human measurements against YOLOv5 predictions. **b** Histogram showing distribution of the differences in the percentage of vitality comparing humans versus YOLOv5

After discovering that the model had less variability than the human measurements, we wanted to test whether the model could be used as a substitution of manual measurements to perform screening of antiparasitic drugs. Therefore, we averaged the percentage of healthy larvae that each human user estimated for each image and then compared the differences with YOLOv5 estimates. We observed that using a Bland-Altman plot to compare the measurements of the model with the human observers, only seven of the estimations were out of the confidence interval (Fig. 7a). Additionally, in a histogram we classified how the differences in the measurements were distributed (Fig. 7b). With an average error of 5.8%, we detected that when estimating the vitality of the images, the model only erred by > 10% in 21 out of 100 images.

**Fig. 8:**
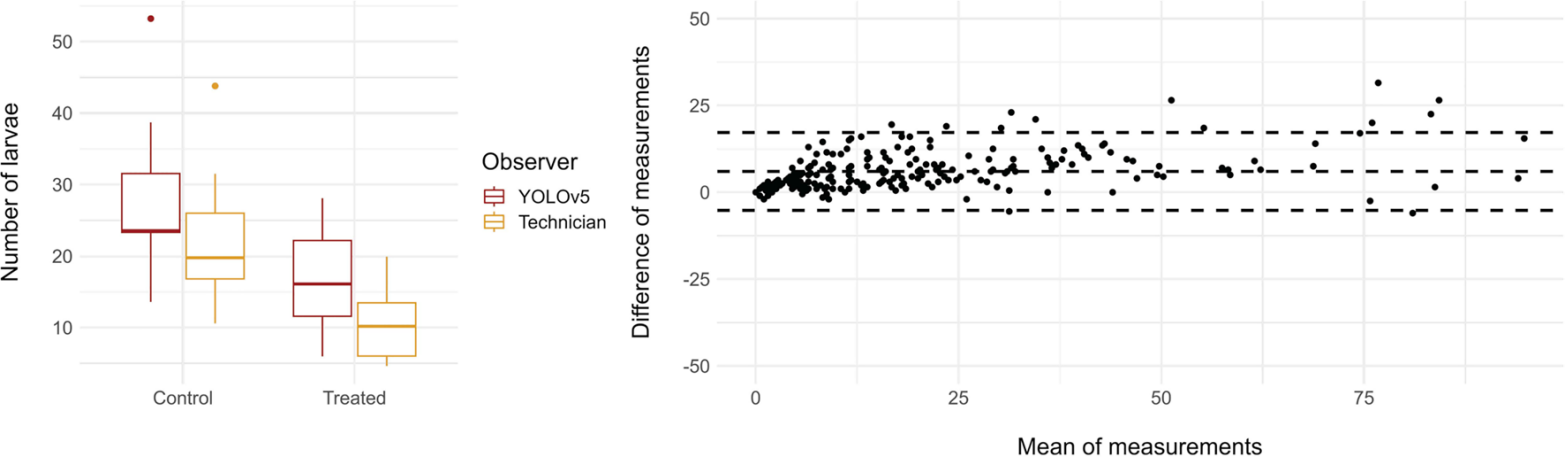
**a** Estimated number of larvae per sample by experienced technicians compared to YOLOv5 model. **b** Bland-Altman plot comparison between the measurements of the experienced technicians and YOLOv5 model

Further application of AxiWorm includes counting the total number of *T. spiralis* larvae in mouse tissue when assaying putative antiparasitic drugs in murine models. In this experiment, larvae are not classified, and only the total number of worms is accounted for. We observed that both measurements gave similar results when counting the number of larvae in the images (Fig. 8a). Furthermore, the statistical analysis by ANOVA indicated that the treatment (*p* = 0.1720), measurement technique (*p* = 0.8322), and interaction between both factors (*p*- = 0.7263) were not significant for explaining the number of larvae counted for each sample. Furthermore, we compared the measurements using a Bland-Altman plot, which shows that most of the measurements were within the confidence interval, with only 12 of the 234 points evaluated falling out of the confidence interval (Fig. 8b).

## Discussion

The conventional method of manual microscopy routinely used in meat inspection at abattoirs, laboratory screening of new molecules, and preclinical studies in animal models of trichinellosis is not only laborious but also subjective. This study introduces a novel approach, called AxiWorm, utilizing image recognition based on deep learning for in vitro drug screening and larva counting in experimentally infected and treated mice. Previous work has shown that larvae exhibit conformational changes induced by different stresses in culture, changing from free-moving states to coiled forms [29]. We believe that this change can be leveraged as a mechanism to infer anti-Trichinella drug activity in culture, which would help screen for active compounds without needing to utilize an extensive number of animals in in vivo assays.

Our model has proven successful to differentiate between healthy and damaged *T. spiralis* larvae, correctly classifying 92% and 98%, respectively, while being unaffected by broken larvae, artifacts, small size objects, low contrast, lighting conditions, or fuzzy objects. Currently, there are published models showing comparable mAP (mean average precision) and precision values. For example, a fruit detection model trained with YOLOv5 resulted in an mAP of 0.893 [30]. Moreover, a model that detects mold on food surfaces showed an mAP of 0.996 [31]. Other models for sperm cell detection, heavy vehicle detection, and sunflower seed classification have also been trained with this object-detection model (YOLOv5), obtaining good mAP values [17, 32, 33].

The AxiWorm system includes image scanning with a smartphone and an inexpensive robotic arm compatible with any standard microscope and operated through a customized app. When paired with the YOLOv5 model that we developed, it took < 2 min to capture, count, and classify a 96-well plate. This drastically reduces work burden compared with manual screening, which can take up to an hour per plate. Our prototype scanner and model demonstrated exceptional utility and efficiency, thus making for a more efficient drug screening assay and increasing the capacity of our laboratory to assay more putative drugs that can become pharmaceutical candidates. Furthermore, the reduction in manual steps in favor of semi-automatic recording of the results reduces human error in the handling and therefore increases the repeatability of the results.

Further experimentation aimed to compare how the model performed against different human observers. Manual counting and classification by experienced technicians showed low consistency, as their coefficient of variation (CV) scores varied significantly between individuals. This indicated that the manual counting method, usually considered the “gold standard,” varied greatly between observers. Meanwhile, our YOLOv5 model CV was significantly lower than for manual counting, thus confirming the reliability of the model and its utility in reducing observer bias and increasing repeatability. Nevertheless, it seems that, from the 300 images that were augmented and evaluated, the model counted and classified two the same and one slightly different, suggesting that one of the modifications that we applied to the images interfered with the model’s ability to properly read the images. This source of variability can be further addressed by re-training the model with a new batch of augmented images. Also, more recent architectures such as YOLOv7, YOLOv8, or YOLOv9 offer improved object detection accuracy, stronger loss functions, and greater efficiency in label assignment and model training [34–36]. Further studies could re-train our dataset with other models and evaluate whether the mistake when adding modifications to the images is avoided during detection. When contrasting the evaluation of the percentage of healthy larvae per well, we observe that both the human observer average (usually considered the gold standard) and the model’s predictions agree on most of the points as per Bland-Altman comparison. This is particularly important when planning to use it as a semi-automatic tool in drug screening assays. Furthermore, when we compare plain differences in the percentage of vitality for each image, we observe that 92% of the images have < 20% error. These levels of error are usually trivial in our screening assays where we usually observe a binomial response in which most larvae are alive or dead at the selected concentration.

Finally, AxiWorm was applied in an in vivo assay to test the number of worms per gram of tissue. The tool successfully counted the total number of larvae in both the control and treatment groups. We are only interested in detecting the quantity of larvae and not classifying them by their viability, as they are only counted and not cultured. Bland-Altman measurements provided comparable results between trained observers and the model when enumerating the total number of larvae in the images. In the pictures, there was some overestimation of the number of larvae due to their poor quality resulting from the larval collection process, which involved issues such as indigestible muscle pieces or broken larvae. However, future studies may enhance accuracy by refining the in vivo collection method or retraining the images with a new model including new YOLO versions in the future just for this purpose, which could have a great impact on the estimation of the number of worms per gram of tissue in other fields such as the meat industry [37, 38]. Overall, this automated system proved to be useful not only with the initial screening of compounds but also in the next step of antiparasitic drug discovery, in vivo assays.

## Conclusions

We have developed AxiWorm, a tool based on AI for in vitro and in vivo assay for drug finding in *T. spiralis* providing an overall balanced model that precisely counts and classifies L1 larvae of *T. spiralis* in pictures, therefore reducing the amount of time spent in the laborious process of drug screening in parasites and reducing the observer bias.

## Data Availability

Data is provided within the manuscript.
